# Fifteen-year trends and differences in mortality rates across sex, age, and race/ethnicity in patients with brainstem tumors

**DOI:** 10.1093/noajnl/vdab137

**Published:** 2021-09-17

**Authors:** Yusuke Tomita, Yoshihiro Tanaka, Nozomu Takata, Elizabeth A Hibler, Rintaro Hashizume, Oren Josh Becher

**Affiliations:** 1 Department of Pediatrics, Northwestern University, Chicago, Illinois, USA; 2 Department of Preventive Medicine, Northwestern University Feinberg School of Medicine, Chicago, Illinois, USA; 3 Center for Arrhythmia Research, Division of Cardiology, Northwestern University Feinberg School of Medicine, Chicago, Illinois, USA; 4 Center for Vascular and Developmental Biology, Feinberg Cardiovascular and Renal Research Institute (FCVRRI), Northwestern University, Chicago, Illinois, USA; 5 Division of Epidemiology, Department of Preventive Medicine, Northwestern University Feinberg School of Medicine, Chicago, Illinois, USA; 6 Division of Hematology-Oncology and Stem Cell Transplant, Ann & Robert H. Lurie Children’s Hospital of Chicago, Chicago, Illinois, USA

**Keywords:** age-adjusted mortality, brainstem glioma, cancer, pediatrics, United States

## Abstract

**Background:**

Localization of tumors to the brainstem carries a poor prognosis, however, risk factors are poorly understood. We examined secular trends in mortality from brainstem tumors in the United States by age, sex, and race/ethnicity.

**Methods:**

We extracted age-adjusted incidence-based mortality rates of brainstem tumors from the Surveillance, Epidemiology, and End Results (SEER) database between 2004 and 2018. Trends in age-adjusted mortality rate (AAMR) were compared by sex and race/ethnicity among the younger age group (0-14 years) and the older age group (>15 years), respectively. Average AAMRs in each 5-year age group were compared by sex.

**Results:**

This study included 2039 brainstem tumor-related deaths between 2004 and 2018. Trends in AAMRs were constant during the study period in both age groups, with 3 times higher AAMR in the younger age group compared to the older age group. Males had a significantly higher AAMR in the older age group, while no racial differences were observed. Intriguingly, AAMRs peaked in patients 5-9 years of age (0.57 per 100 000) and in patients 80-84 years of age (0.31 per 100 000), with lower rates among middle-aged individuals. Among 5-9 years of age, the average AAMR for females was significantly higher than that of males (*P* = .017), whereas the reverse trend was seen among those 50-79 years of age.

**Conclusions:**

Overall trends in AAMRs for brainstem tumors were constant during the study period with significant differences by age and sex. Identifying the biological mechanisms of demographic differences in AAMR may help understand this fatal pathology.

**Key Points**

- Trends in AAMR of brainstem tumors were constant across age-sex groups.
- AAMR was significantly higher in the younger age group than the older age group.
- AAMR peaked in the 5-9 age group, with a significantly higher AAMR in females in that age group.

**Importance of the Study**

Localization of tumors to the brainstem still carries a poor prognosis, even though rigorous clinical research has been conducted. However, the mortality rate in brainstem tumors has not been well described. In this study, we conducted a comprehensive characterization of the mortality rate of this heterogeneous group of tumors in the United States with the Surveillance, Epidemiology, and End Results (SEER) database to investigate the trends in age-adjusted mortality rate (AAMR) by age, sex, and race/ethnicity. Overall trends in AAMR of brainstem tumors were constant during the study period, suggesting that brainstem tumors remained a catastrophic pathology regardless of age. Also, among those 5-9 years of age, the average AAMR in females was significantly higher than that in males, probably because of the higher age-adjusted incidence rates (AAIRs). Identifying the causes of demographic differences in AAMR may improve our understanding of brainstem tumors.

Brainstem tumors are often fatal and account for 10%-20% of all central nervous system tumors in the pediatric population.^1^ Diffuse intrinsic pontine glioma (DIPG) are infiltrative tumors that arise in the pons and the most aggressive subtype of brainstem tumors. DIPG accounts for approximately 75% of pediatric brainstem tumors with a median survival of 4-15 months.^1,2^ By comparison, the prognosis of adult DIPG ranges from 30 to 40 months, although adult brainstem tumors, which are less common than childhood DIPG, vary in their biological and molecular features.^3^ Despite rigorous clinical research,^4–7^ prognosis has not improved for patients with DIPG.^8^

Mortality rate, which serves as a surrogate of disease severity and treatment effectiveness over time, is a useful parameter for evaluating progress in improving the average prognosis for brainstem tumors. However, the mortality rate in brainstem tumors has not been quantitatively evaluated, and demographic differences in mortality rates have not been reported. It is vital to examine the mortality rate of brainstem tumors and how it differs between patient groups. Zhang et al have reported an impact of socioeconomic status on the survival of colorectal cancer patients.^9^ Recently, Ostrom et al reported that race/ethnicity impacted the time for the initiation of radiotherapy and chemotherapy among glioma patients.^10^ Therefore, investigating demographic and racial disparities in the mortality rates of brainstem tumors might identify a novel prognostic factor in patients with these refractory tumors.

The Surveillance, Epidemiology, and End Results (SEER) database comprehensively collects cancer vital statistics from population-based registries. The database is a valuable resource that has been used in numerous cancer epidemiology studies.^11,12^ The large sample size of SEER allows investigation of mortality rate and subgroup analyses by baseline characteristics of rare cancers. Therefore, we aimed to quantify the trends in mortality related to brainstem tumors and examine demographic differences using the available data in SEER.

## Materials and Methods

This study utilized de-identified data from the SEER database, the publicly available cancer dataset maintained by the National Cancer Institute covering approximately 34.6% of the US population (https://seer.cancer.gov/data/). Data on demographic data, primary tumor site, tumor morphology, stage at diagnosis, the first course of treatment, and follow-up data on vital status are available in the dataset. This secondary analysis of de-identified data was reviewed by the Northwestern University Human Subjects Protection program.

We queried cancer statistics from SEER*Stat software version 8.3.9 (Information Management Service, Inc., Calverton, MD, USA) between January 1, 2004 and December 31, 2018. The study period, especially starting year, was determined based on the following reasons; first, in the previous report, authors extracted low-grade brain stem glioma cases from 2004 to 2015. Second, no new chemotherapeutic reagent for malignant gliomas has prolonged overall survival from 2004, when the efficacy of concurrent use of temozolomide and radiation therapy was confirmed.^13^ We extracted the age-adjusted incidence and incidence-based mortality rate data of all types of brainstem tumors (Primary Site-labeled, C71.7-Brain stem) only with malignant behaviors. Additionally, we also extracted an annual number of brainstem tumor cases, annual population estimates, age at diagnosis, age at death, sex, race/ethnicity, tumor types, and histology of lesion if available. Although US cancer mortality data based on death certificates are frequently utilized, there is a limitation that this measure fails to include the data on the onset of diseases such as year-of-diagnosis, age at diagnosis, stage of disease at diagnosis, and histology of lesions. In contrast, population-based cancer registries collect these types of data and allow the calculation of an incidence-based mortality rate. This incidence-based mortality rate may allow for evaluation of factors that may contribute to mortality associated with the cancer onset, and it is noted that the incidence-based mortality rate is different from the mortality rate based on death certificates.^14^

Supplementary Table 1 shows the tumor types analyzed in this study, classified using the International Classification of Diseases for Oncology, Third Edition. Approximately half of the cases (51.4%) were histologically confirmed, and the rest of half cases were diagnosed radiologically or clinically. Also, we extracted age-adjusted mortality rates (AAMRs) of histologically confirmed high-grade glioma cases (ICD-O-3 codes: 9380, 9385, 9400, 9401, 9440, 9441, 9442, 9451, 9460). Annual population estimates (denominators) between 2004 and 2018 were obtained from SEER*Stat Software (https://seer.cancer.gov/popdata/l). The number of decedents and the population (denominators) used for each analysis were shown in Supplementary Tables 2–8.

To evaluate the trend of AAMR, we divided the cohort into the younger age groups (0-14 years) and the older age groups (15-85+ years), and we compared AAMRs by sex and race/ethnicity. Race/ethnicity was categorized as non-Hispanic Black, non-Hispanic White, and Hispanic.^15^ Average AAMRs in each 5-year age group were compared by sex, except age 0 which was separately classified. To compare the age distribution of AAMRs as well as incidence rates, we separately extracted the age-adjusted incidence rates (AAIRs) in each 5-year age group using SEER*stat. AAIRs were filtered by the same information as AAMRs, and by having the case-specific death classification as an endpoint. The number of patients diagnosed as having brainstem tumors and the population (denominators) used for AAMRs and AAIRs were shown in Supplementary Tables 9 and 10.

### Statistics

We computed AAMRs per 100 000 population using yearly population estimates standardized to the US population (https://seer.cancer.gov/stdpopulations/) in the year 2000. We examined trends over time estimating average annual percent change (AAPC) and identified up to 1 inflection point using the Joinpoint Regression Program (National Cancer Institute), which calculates AAPC with 95% confidence interval (CI) and statistical differences between each regression estimate.^16^ We also examined trends in AAMR over time between age groups (younger: 0-14 years, older: ≥15 years) then stratified further by race/ethnicity and sex. We also analyzed AAMRs and AAIRs by 5-year age groups and sex. The rate ratio was calculated and compared with SEER*Stat software to assess differences in AAMRs and AAIRs between females and males. All the figures were generated with GraphPad Prism (version 9.1.0).

## Results

Overall, the present study included 2039 decedents [younger age group, 838 (41.1%); males, 1076 (52.8%)] diagnosed with brainstem tumors between 2004 and 2018. The AAMRs data in this study were collected from age groups ranging from 0 to 85+ years. As expected, some tumor types were primarily observed only in the pediatric setting, such as atypical teratoid/rhabdoid tumors, while others were primarily noted in the adult setting such as lymphomas (Supplementary Table 1).

**Figure 1.**
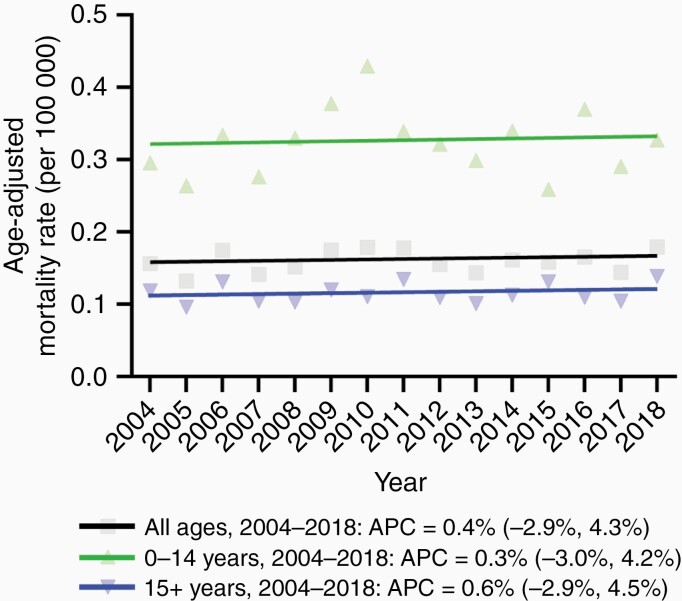
Trends in age-adjusted mortality rate related to brainstem tumors per 100 000 by age between 2004 and 2018. Age-adjusted mortality rates are summarized as a mean value.

Trends in AAMR in the two age groups over time are shown in Figure 1 and Table 1. AAMRs in both age groups were constant without any significant inflection point. AAMR in the younger group was significantly higher than that in the older group. Although the AAMR in the younger age group fluctuated, ranging from 0.29 to 0.43 per 100 000 between 2004 and 2018, Joinpoint regression did not identify any significant inflection points between 2004 and 2018. The AAMR was almost unchanged over time both among the younger group (AAPC: 0.3 [95% CI: −3.0 to 4.2]; Table 1), and among the older group (AAPC: 0.6 [95% CI: −2.9 to 4.5], Table 1). Similar trends of AAMR were also observed in the high-grade gliomas in the brainstem (Supplementary Figure 1).

**Table 1.** Trends in Age-Adjusted Mortality in Brainstem Tumors by Age Groups

	Overall(N = 2039)		Younger (≤14 yr)(N = 838)		Older (≥15 yr)(N = 1201)	
Year	AAMR (95% CI)	AAPC (95% CI)	AAMR (95% CI)	AAPC (95% CI)	AAMR (95% CI)	AAPC (95% CI)
2004	0.16 (0.13, 0.19)	0.4 (−2.9, 4.3)	0.30 (0.22, 0.39)	0.3 (−3.0, 4.2)	0.12 (0.09, 0.15)	0.6 (−2.9, 4.5)
2005	0.13 (0.11, 0.16)		0.26 (0.19, 0.35)		0.10 (0.07, 0.12)	
2006	0.17 (0.15, 0.21)		0.33 (0.25, 0.43)		0.13 (0.10, 0.16)	
2007	0.14 (0.12, 0.17)		0.28 (0.20, 0.37)		0.10 (0.08, 0.13)	
2008	0.15 (0.13, 0.18)		0.33 (0.25, 0.43)		0.10 (0.08, 0.13)	
2009	0.18 (0.15, 0.21)		0.38 (0.29, 0.48)		0.12 (0.09, 0.15)	
2010	0.18 (0.15, 0.21)		0.43 (0.34, 0.54)		0.11 (0.09, 0.14)	
2011	0.18 (0.15, 0.21)		0.34 (0.26, 0.44)		0.13 (0.11, 0.17)	
2012	0.16 (0.13, 0.18)		0.32 (0.24, 0.42)		0.11 (0.09, 0.14)	
2013	0.14 (0.12, 0.17)		0.30 (0.22, 0.39)		0.10 (0.08, 0.13)	
2014	0.16 (0.14, 0.19)		0.34 (0.26, 0.44)		0.11 (0.09, 0.14)	
2015	0.16 (0.13, 0.19)		0.26 (0.19, 0.35)		0.13 (0.11, 0.16)	
2016	0.17 (0.14, 0.20)		0.37 (0.29, 0.47)		0.12 (0.09, 0.14)	
2017	0.14 (0.12, 0.17)		0.29 (0.22, 0.38)		0.10 (0.08, 0.13)	
2018	0.18 (0.15, 0.21)		0.33 (0.25, 0.43)		0.14 (0.11, 0.17)	

AAMR, age-adjusted mortality rate; AAPC, average annual percent change; CI, confidence interval.

**Figure 2.**
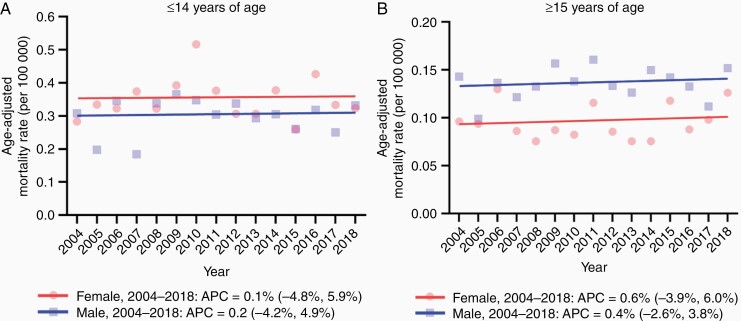
Trends in age-adjusted mortality rate related to brainstem tumors per 100 000 by age and sex between 2004 and 2018. (A) Trends in age-adjusted mortality rate by sex in younger (≤14 years) age group. (B) Trends in age-adjusted mortality rate by sex in older (≥15 years) age group. Age-adjusted mortality rates are summarized as a mean value.

Figure 2 shows trends in AAMR by sex across the two age groups. Among the younger group, females tended to have higher AAMRs over time compared with males, which did not reach a statistical significance (Figure 2A). In contrast, males had consistently higher AAMRs compared to females in the older group over the study period (Figure 2B). AAMR in older male over the 15 years was 0.14 (95% CI: 0.13-0.15), which was significantly higher than that in female (0.10 [95% CI: 0.09-0.10], *P* < .001). In the younger group, the AAMR in both females and males did not change significantly from 2004 to 2018 (Figure 2A; Table 2). Similarly, in the older group, AAMRs in both males and females did not change significantly between 2004 and 2018 (Figure 2B; Table 2). Trends of AAMR by sex across the two age groups were also similar in the high-grade gliomas in the brainstem (Supplementary Figure 2A and B).

**Table 2.** Trends in Age-Adjusted Mortality Rate in Brainstem Tumors by Age and Sex Groups

	Younger (≤14 yr)				Older (≥15 yr)			
	Females (N = 395)		Males (N = 443)		Females (N = 520)		Males (N = 681)	
	AAMR (95% CI)	AAPC (95% CI)	AAMR (95% CI)	AAPC (95% CI)	AAMR (95% CI)	AAPC (95% CI)	AAMR (95% CI)	AAPC (95% CI)
2004	0.28 (0.18, 0.42)	0.1 (−4.8, 5.9)	0.31 (0.20, 0.45)	0.2 (−4.2, 4.9)	0.10 (0.07, 0.14)	0.6 (−3.9, 6.0)	0.14 (0.10, 0.19)	−0.4 (−2.6, 3.8)
2005	0.33 (0.22, 0.48)		0.20 (0.12, 0.32)		0.09 (0.06, 0.13)		0.10 (0.07, 0.14)	
2006	0.32 (0.21, 0.47)		0.35 (0.23, 0.49)		0.13 (0.09, 0.17)		0.14 (0.10, 0.19)	
2007	0.37 (0.25, 0.53)		0.18 (0.11, 0.30)		0.09 (0.06, 0.12)		0.12 (0.09, 0.17)	
2008	0.32 (0.21, 0.47)		0.34 (0.23, 0.48)		0.08 (0.05, 0.11)		0.13 (0.10, 0.18)	
2009	0.39 (0.27, 0.55)		0.37 (0.25, 0.51)		0.09 (0.06, 0.13)		0.16 (0.12, 0.21)	
2010	0.52 (0.38, 0.69)		0.35 (0.24, 0.49)		0.08 (0.06, 0.12)		0.14 (0.10, 0.18)	
2011	0.38 (0.26, 0.53)		0.30 (0.20, 0.44)		0.12 (0.08, 0.15)		0.16 (0.12, 0.21)	
2012	0.31 (0.20, 0.45)		0.34 (0.23, 0.48)		0.09 (0.06, 0.12)		0.13 (0.10, 0.18)	
2013	0.31 (0.20, 0.45)		0.29 (0.19, 0.43)		0.07 (0.05, 0.11)		0.13 (0.09, 0.17)	
2014	0.38 (0.26, 0.53)		0.31 (0.20, 0.44)		0.08 (0.05, 0.11)		0.15 (0.11, 0.20)	
2015	0.26 (0.16, 0.39)		0.26 (0.17, 0.39)		0.12 (0.09, 0.16)		0.14 (0.10, 0.19)	
2016	0.43 (0.30, 0.59)		0.32 (0.21, 0.46)		0.09 (0.06, 0.12)		0.13 (0.10, 0.18)	
2017	0.33 (0.22, 0.48)		0.25 (0.16, 0.38)		0.09 (0.06, 0.13)		0.10 (0.08, 0.15)	
2018	0.32 (0.21, 0.47)		0.33 (0.22, 0.48)		0.13 (0.09, 0.17)		0.15 (0.11, 0.20)	

AAMR, age-adjusted mortality rate; AAPC, average annual percent change; CI, confidence interval.

**Figure 3.**
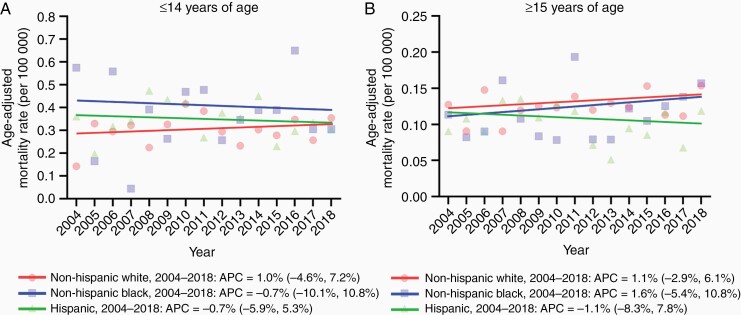
Trends in age-adjusted mortality rate related to brainstem tumors per 100 000 by race/ethnicity across age groups between 2004 and 2018. (A) Trends in age-adjusted mortality rate by race/ethnicity in younger (≤14 years) age group. (B) Trends in age-adjusted mortality rate by race/ethnicity in older (≥15 years) age group. Age-adjusted mortality rates are summarized as a mean value.

Differences in AAMR from 2004 to 2018 by sex and race/ethnicity are shown in Figure 3 and Supplementary Table 11. Among younger age, the non-Hispanic White group had a relatively lower AAMR compared with those in the non-Hispanic Black and Hispanic groups (Figure 3A). Among older age, in contrast, the Hispanic group had a slightly lower AAMR compared with those in the non-Hispanic White and Black groups (Figure 3B). Notably, over the 15-year study period, AAMRs remained unchanged for all age and race/ethnicity subgroups (Supplementary Table 11).

**Figure 4.**
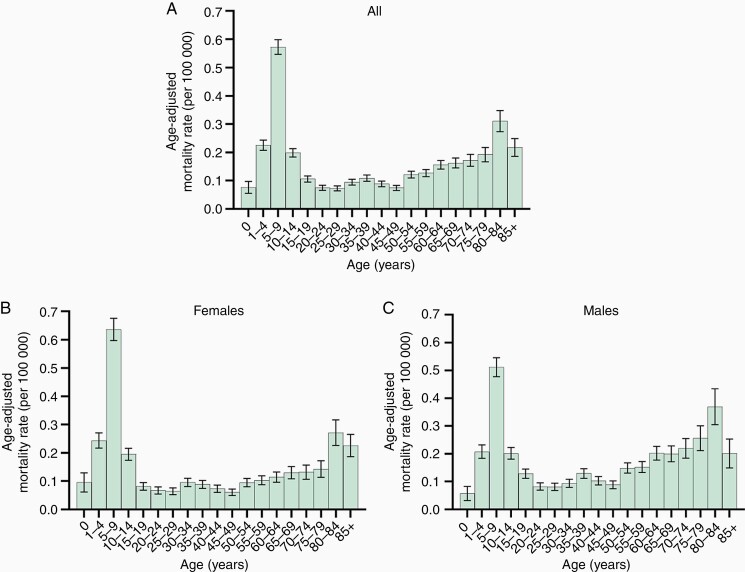
Distribution of age-adjusted mortality rate related to brainstem tumors per 100 000 for each 5-year age group. Histogram showing age-adjusted mortality rate for each age group among females and males (A), females (B), and males (C). Age-adjusted mortality rates are summarized as mean with standard error.

Figure 4 and Supplementary Table 12 demonstrate the differences in average AAMRs by 5-year age groups. Overall, the average AAMR was biphasic, with one sharp and large peak in the 5- to 9-year-old group (average AAMR 0.57, 95% CI: 0.52-0.63), and another broad and smaller peak in the 80- to 84-year-old group (average AAMR: 0.31, 95% CI: 0.24-0.39, Figure 4A). The first peak was significantly lower in males (average AAMR: 0.51 [95% CI: 0.45-0.58] compared with females (average AAMR 0.64 [95% CI: 0.56-0.72], *P* = .017). This was reversed in the second peak, which was higher in males (average AAMR 0.37 [95% CI: 0.25-0.52]) than in females (average AAMR 0.27 [95% CI: 0.19-0.38]) with no statistical significance (Figure 4B and C; Supplementary Table 12). Average AAMRs in males were significantly higher than those in females in the 50- to 54-, 60- to 64-, and 75- to 79-year-old group (*P* = .005, *P* = .005, and *P* = .039, respectively; Supplementary Table 3).

To investigate if these age distributions of AAMRs were related to the incidence rates, we extracted the average AAIRs by the 5-year age group (Supplementary Table 13). The first peak consisted of two 5-year age groups; 1- to 4-year-old group (average AAIR 0.35 [95% CI: 0.31-0.40] and 5- to 9-year-old group (average AAIR 0.43 [95% CI: 0.39-0.48]). Interestingly, average AAIRs in male was substantially lower than females in the 1- to 4-year-old group (*P* = .051), although not in the 5- to 9-year-old group (*P* = .296).

Finally, as the brainstem gliomas account for majority of the brainstem tumors, we extracted the average AAMR and AAIRs of brainstem gliomas by the 5 age groups (Supplementary Figure 3A–C). Average AAMRs in male was significantly lower than females both in the 1- to 4-year-old group (male 0.11 [95% CI: 0.08-0.15], female 0.19 [95% CI: 0.15-0.25], *P* = .010) and in the 5- to 9-year-old group (male 0.43 [95% CI: 0.37-0.50], female 0.56 [95% CI: 0.49-0.64], *P* = .010) (Supplementary Table 14). Interestingly, average AAIRs in male was significantly lower than females in the 1- to 4-year-old group (male 0.21 [95% CI: 0.16-0.26], female 0.32 [95% CI: 0.26-0.38], *P* = .006), but not in the 5- to 9-year-old group (male 0.36 [95% CI: 0.31-0.42], female 0.42 [95% CI: 0.36-0.49], *P* = .166) (Supplementary Table 15).

## Discussion

Although previous studies have highlighted trends in the incidence of brainstem tumors, this is the first report of AAMRs in brainstem tumors. Here, we showed stable trends in AAMR related to brainstem tumors in a recent 15-year period. Additionally, we revealed demographic differences in AAMRs related to brainstem tumors. Our findings underscore the urgent need for further research and development of effective treatments to reduce mortality associated with brainstem tumors.

In the younger age group (0-14 years), our analysis showed constant AAMRs. Given that the incidence rate of brainstem tumors is also reportedly constant in the younger age group,^2^ the constant case fatality rate during the same period suggests that brainstem tumors remain a lethal pathology without effective treatments even in the modern era. In fact, no clinical trials have shown improvement in the prognosis of brainstem tumors.^8^ In contrast, AAMRs in the older age group (15 years of age and older) were significantly lower than those in the younger age group. One plausible explanation for the disproportionate AAMRs between the older and younger age groups is the lower incidence rate of brainstem tumors and brainstem tumor heterogeneity in older patients.^3,17,18^ Nevertheless, the trend in AAMR was constant in both the older and younger age groups in our study, indicating that brainstem tumors remain a catastrophic pathology with poor prognosis regardless of age. Also, the constant trend in AAMR indicated that the most likely cause of the fluctuation in AAMR is the fluctuation in the number of patients diagnosed as having brainstem tumors.

We found that AAMRs were higher in females than those in males within the 5- to 9-year-old age group, whereas AAIRs were higher in females than those in males within the 1- to 4-year-old age group. These findings suggest that the higher AAIR in females within the 1- to 4-year-old age group is attributed to the higher AAMR in females within the 5- to 9-year-old age group. This finding is supported by a recent publication. Patil et al have recently conducted an analysis of high-grade brainstem gliomas with Central Brain Tumor Registry of the United States (CBTRUS), in which the overall AAIR was higher in females.^19^ The other plausible explanation is that the SEER database might contain certain DIPG subtypes such as DIPG with *ACVR1* (activin A receptor type 1) mutation at this age group, which has a predominance of a female with a 1.75:1 female to male ratio.^20,21^ Therefore, the higher AAMR in females relative to males within the 5-9 age group is likely attributed to the higher incidence rate in females.^19^

In the older ages, AAMRs were higher in males than in females, especially within the 50- to 69-year-old age groups, and had a gradual peak in the 80- to 84-year-old age group among both sexes. Previous studies have reported that adult gliomas including brainstem gliomas are male-dominant,^22,23^ which is consistent with our observation of higher AAMRs in older males compared to younger females. Elderly patients including the age group of 80-84 years are more likely to have tumors than younger patients given that the incidence of malignant tumors increases with aging (Supplementary Tables 13 and 15). In addition, in clinical settings, elderly patients are less likely to tolerate standard treatment due to the higher rate of comorbidities and low-performance status. As a result, elderly patients are considered to have higher risks of mortality.

Our data illustrated no significant differences in AAMR by race/ethnicity in either the younger or older age groups. Our findings, using a large cohort involving several registries that comprehensively captures deaths in cancer patients, are consistent with those of a previous population-based study with the SEER database.^24^ In the study, they analyzed the demographics, tumor characteristics, and survival analysis of 154 high-grade brainstem glioma, showing that race/ethnicity was not a predictor of cancer-specific mortality.^24^ In contrast, Patil et al have recently conducted the survival analysis of brainstem glioma using CBTRUS and National Program of Cancer Registries (NPCR), showing a significant difference in the risk of death. In the study, they extracted 4486 patients with primary malignant high-grade brainstem gliomas, and Cox proportional hazard regression models showed that Blacks had a higher risk of death compared to Whites (HR: 1.19, 95% CI: 1.07-1.33; *P* = .002).^19^ One of the plausible explanations regarding this point is that our study population was smaller and hence underpowered to detect significant racial differences in AAMR. Also, we cannot exclude out the possibility that we may have underestimated the AAMR among Hispanic patients due to misclassification.^25,26^ Nevertheless, SEER is considered as a unique data source that is sufficiently large to allow exploration of AAMRs in rare types of cancer including examination of racial disparities in AAMRs

Our data showed that trends in AAMR in the younger age group slightly declined, especially for males. In general, AAMR is determined by a balance between incidence and case fatality rate. According to the CBTRUS database, the age-adjusted incidence of primary central nervous system tumors is increasing among younger age groups.^22^ As for the case fatality rate, numerous clinical trials with chemotherapeutic agents have failed to prolong survival in patients diagnosed with DIPG, one of the most common brain tumors in pediatrics.^8^ Collectively, AAMR in younger males appears to have unexpectedly declined over the past 10 years. Therefore, our study’s AAMR decline is likely to represent random variation or changes in underlying demographics rather than a real change.

### Limitations

The strengths of this study include the use of up-to-date data on brainstem tumor mortality between 2004 and 2018 and the use of large nationwide registry data covering approximately one-third of the US population. Therefore, we can generalize the present observations, and the results are likely to be less biased compared to previous observational studies or single-center registries. The large sample size also allowed us to conduct several subgroup analyses to examine detailed mortality trends in brainstem tumors. This study has several limitations, however. First, the study did not consider important confounding factors, such as frailty, functional status, comorbidity, medications, or treatment-related information such as surgery with or without chemotherapy. Although we performed several subgroup analyses, the effects of residual confounding should be considered when interpreting the results. Second, this study was conducted in the United States including mainly non-Hispanic Black and White individuals, which limits the generalizability of our results to other populations in different countries. Third, the present study investigated the average trends in AAMR of brainstem tumors. Brainstem tumors are a very heterogeneous group of tumors including both benign and highly malignant tumors and are classified based on their anatomical location, histopathology, and molecular features.^1,27^ Therefore, the SEER database would provide more useful information if molecular features of the tumors such as H3K27M status were analyzed and collected on each case. Forth, the results in this study should be interpreted with caution because factors like lead-time bias can influence the analyses based on incidence-based mortality. Finally, miscoding or misclassification is likely to have occurred, which can affect the results of our analyses.^25,26^

## Conclusions

Overall trends in AAMR related to brainstem tumors were constant between 2004 and 2018, suggesting that brainstem tumors including gliomas remain a lethal constellation of neoplasms without effective treatments in the modern era. We also found age and sex differences in brainstem tumor AAMRs. The high AAMR in females in the younger ages is likely due to the higher incidence rate in females than that in males. Identifying the causes of demographic differences in AAMR may help better understand this fatal pathology.

## Supplementary Material

vdab137_suppl_Supplementary_File
