# Supplementary material for: Fifteen-year trends and differences in mortality rates across sex, age, and race/ethnicity in patients with brainstem tumors
Source: Neurooncol Adv. 2021 Sep 17;3(1):vdab137. doi: 10.1093/noajnl/vdab137 (PMC8528263; doi:10.1093/noajnl/vdab137)

**Supplemental Table 1. Distribution of brainstem tumors of all the decedents included in this analysis: SEER, 2004-2018.**

| **IDC-O code** | **Tumor type** | **Number of Decedents (n)** | | |
| --- | --- | --- | --- | --- |
|  |  | **Total** | **Aged 14 and younger** | **Aged 15 and older** |
| 8000/3 | Neoplasm, malignant | 68 | 10 | 58 |
| 8001/3 | Tumor cells, malignant | 2 | 1 | 1 |
| 8070/3 | Squamous cell carcinoma, NOS | 1 | 0 | 1 |
| 8720/3 | Malignant melanoma, NOS | 1 | 0 | 1 |
| 8810/3 | Fibrosarcoma, NOS | 1 | 0 | 1 |
| 8991/3 | Embryonal sarcoma | 1 | 1 | 0 |
| 9064/3 | Germinoma | 3 | 0 | 3 |
| 9070/3 | Embryonal carcinoma, NOS | 1 | 1 | 0 |
| 9081/3 | Teratocarcinoma | 1 | 1 | 0 |
| 9085/3 | Mixed germ cell tumor | 1 | 0 | 1 |
| 9150/3 | Hemangiopericytoma, malignant | 1 | 0 | 1 |
| 9370/3 | Chordoma, NOS | 4 | 0 | 4 |
| 9380/3 | Glioma, malignant | 990 | 521 | 469 |
| 9381/3 | Gliomatosis cerebri | 4 | 0 | 4 |
| 9382/3 | Mixed glioma | 9 | 2 | 7 |
| 9385/3 | Diffuse intrinsic pontine glioma, H3 K27M-mutant | 7 | 5 | 2 |
| 9390/3 | Choroid plexus papilloma, malignant | 1 | 1 | 0 |
| 9391/3 | Ependymoma, NOS | 116 | 23 | 93 |
| 9392/3 | Ependymoma, anaplastic | 33 | 21 | 12 |
| 9393/3 | Papillary ependymoma, NOS | 2 | 0 | 2 |
| 9400/3 | Astrocytoma, NOS | 164 | 44 | 120 |
| 9401/3 | Astrocytoma, anaplastic | 121 | 43 | 78 |
| 9411/3 | Gemistocytic astrocytoma | 1 | 0 | 1 |
| 9420/3 | Fibrillary astrocytoma | 19 | 5 | 14 |
| 9421/3 | Pilocytic astrocytoma, malignant | 76 | 17 | 59 |
| 9424/3 | Pleomorphic xanthoastrocytoma | 1 | 0 | 1 |
| 9440/3 | Glioblastoma, NOS | 230 | 64 | 166 |
| 9441/3 | Giant cell glioblastoma | 2 | 0 | 2 |
| 9442/3 | Gliosarcoma | 2 | 0 | 2 |
| 9450/3 | Oligodendroglioma, NOS | 3 | 0 | 3 |
| 9451/3 | Oligodendroglioma, anaplastic | 5 | 1 | 4 |
| 9470/3 | Medulloblastoma, NOS | 52 | 29 | 23 |
| 9471/3 | Desmoplastic nodular medulloblastoma | 1 | 0 | 1 |
| 9473/3 | Primitive neuroectodermal tumor | 23 | 19 | 4 |
| 9474/3 | Large cell medulloblastoma | 8 | 6 | 2 |
| 9478/3 | Embryonal tumor with multilayered rosettes with  C19MC alteration | 1 | 1 | 0 |
| 9490/3 | Ganglioneuroblastoma | 1 | 0 | 1 |
| 9500/3 | Neuroblastoma, NOS | 1 | 0 | 1 |
| 9508/3 | Atypical teratoid/rhabdoid tumor | 23 | 22 | 1 |
| 9530/3 | Meningioma, malignant | 2 | 0 | 2 |
| 9590/3 | Malignant lymphoma, NOS | 15 | 0 | 15 |
| 9591/3 | Non-Hodgkin lymphoma, NOS | 9 | 0 | 9 |
| 9675/3 | Malig lymphoma, mixed small & large cell, diffuse (OBS 2010+) see 9690/3 | 1 | 0 | 1 |
| 9680/3 | Diffuse large B-cell lymphoma (DLBCL), NOS | 29 | 0 | 29 |
| 9687/3 | Burkitt lymphoma | 1 | 0 | 1 |
| 9702/3 | Peripheral T-cell lymphoma, NOS | 1 | 0 | 1 |

**Supplemental Table 2. Trends in number of decedents and population across age groups**

|  | **All (N = 2039)** | | **Younger (≤ 14 years) (N = 838)** | | **Older (≥ 15 years) (N =1201)** | |
| --- | --- | --- | --- | --- | --- | --- |
| Year | Descents | Population | Descents | Population | Descents | Population |
| 2004 | 126 | 82,055,585 | 51 | 17,631,428 | 75 | 64,424,157 |
| 2005 | 107 | 82,537,476 | 45 | 17,503,724 | 62 | 65,033,752 |
| 2006 | 143 | 83,099,557 | 57 | 17,404,381 | 86 | 65,695,176 |
| 2007 | 117 | 83,810,676 | 47 | 17,402,455 | 70 | 66,408,221 |
| 2008 | 126 | 84,618,783 | 57 | 17,448,105 | 69 | 67,170,678 |
| 2009 | 146 | 85,402,713 | 65 | 17,476,352 | 81 | 67,926,361 |
| 2010 | 151 | 86,139,591 | 75 | 17,500,122 | 76 | 68,639,469 |
| 2011 | 151 | 86,790,832 | 59 | 17,486,158 | 92 | 69,304,674 |
| 2012 | 133 | 87,404,830 | 56 | 17,447,725 | 77 | 69,957,105 |
| 2013 | 124 | 87,981,722 | 52 | 17,419,428 | 72 | 70,562,294 |
| 2014 | 141 | 88,585,507 | 59 | 17,392,953 | 82 | 71,192,554 |
| 2015 | 139 | 89,189,512 | 45 | 17,348,017 | 94 | 71,841,495 |
| 2016 | 147 | 89,764,615 | 64 | 17,313,794 | 83 | 72,450,821 |
| 2017 | 127 | 90,244,431 | 50 | 17,281,852 | 77 | 72,962,579 |
| 2018 | 161 | 90,571,210 | 56 | 17,194,763 | 105 | 73,376,447 |

**Supplemental Table 3. Trends in the number of decedents from brainstem tumors and estimated number of population by sex groups across age groups**

| **Younger (≤ 14 years)** | | | | |
| --- | --- | --- | --- | --- |
|  | **Female (N = 443)** | | **Male (N =395)** | |
| Year | Descents | Population | Descents | Population |
| 2004 | 24 | 8,610,981 | 27 | 9,020,447 |
| 2005 | 28 | 8,550,306 | 17 | 8,953,418 |
| 2006 | 27 | 8,503,488 | 30 | 8,900,893 |
| 2007 | 31 | 8,500,992 | 16 | 8,901,463 |
| 2008 | 27 | 8,525,325 | 30 | 8,922,780 |
| 2009 | 33 | 8,541,776 | 32 | 8,934,576 |
| 2010 | 44 | 8,555,469 | 31 | 8,944,653 |
| 2011 | 32 | 8,551,163 | 27 | 8,934,995 |
| 2012 | 26 | 8,534,232 | 30 | 8,913,493 |
| 2013 | 26 | 8,521,981 | 26 | 8,897,447 |
| 2014 | 32 | 8,508,961 | 27 | 8,883,992 |
| 2015 | 22 | 8,487,571 | 23 | 8,860,446 |
| 2016 | 36 | 8,470,470 | 28 | 8,843,324 |
| 2017 | 28 | 8,453,097 | 22 | 8,828,755 |
| 2018 | 27 | 8,407,935 | 29 | 8,786,828 |

| **Older (≥ 15 years)** | | | | |
| --- | --- | --- | --- | --- |
|  | **Female (N = 520)** | | **Male (N =681)** | |
| Year | Descents | Population | Descents | Population |
| 2004 | 43 | 31,466,308 | 32 | 32,957,849 |
| 2005 | 30 | 31,776,868 | 32 | 33,256,884 |
| 2006 | 42 | 32,112,513 | 44 | 33,582,663 |
| 2007 | 40 | 32,459,764 | 30 | 33,948,457 |
| 2008 | 43 | 32,838,068 | 26 | 34,332,610 |
| 2009 | 51 | 33,215,002 | 30 | 34,711,359 |
| 2010 | 47 | 33,557,207 | 29 | 35,082,262 |
| 2011 | 50 | 33,887,208 | 42 | 35,417,466 |
| 2012 | 46 | 34,220,961 | 31 | 35,736,144 |
| 2013 | 44 | 34,525,121 | 28 | 36,037,173 |
| 2014 | 51 | 34,845,245 | 31 | 36,347,309 |
| 2015 | 49 | 35,177,435 | 45 | 36,664,060 |
| 2016 | 49 | 35,485,515 | 34 | 36,965,306 |
| 2017 | 41 | 35,744,740 | 36 | 37,217,839 |
| 2018 | 55 | 35,950,905 | 50 | 37,425,542 |

**Supplemental Table 4. Trends in number of decedents and population by race groups across age groups**

| **Younger (≤ 14 years)** | | | | | | |
| --- | --- | --- | --- | --- | --- | --- |
|  | **NHB (N = 130)** | | **NHW (N = 352)** | | **Hispanic (N =278)** | |
| Year | Descents | Population | Descents | Population | Descents | Population |
| 2004 | 12 | 8,631,169 | 14 | 2,457,988 | 17 | 4,951,165 |
| 2005 | 27 | 8,461,564 | 4 | 2,403,020 | 10 | 5,031,574 |
| 2006 | 24 | 8,310,466 | 13 | 2,362,362 | 16 | 5,107,093 |
| 2007 | 26 | 8,202,610 | 1 | 2,359,067 | 17 | 5,195,998 |
| 2008 | 18 | 8,116,260 | 9 | 2,353,504 | 25 | 5,304,586 |
| 2009 | 26 | 8,025,076 | 6 | 2,347,688 | 23 | 5,404,521 |
| 2010 | 33 | 7,947,007 | 11 | 2,343,786 | 23 | 5,489,234 |
| 2011 | 30 | 7,877,329 | 11 | 2,339,649 | 15 | 5,537,362 |
| 2012 | 23 | 7,807,016 | 6 | 2,330,107 | 21 | 5,565,027 |
| 2013 | 18 | 7,741,753 | 8 | 2,321,365 | 20 | 5,590,147 |
| 2014 | 23 | 7,679,724 | 9 | 2,315,703 | 25 | 5,610,761 |
| 2015 | 21 | 7,610,313 | 9 | 2,305,497 | 13 | 5,622,121 |
| 2016 | 26 | 7,550,096 | 15 | 2,300,473 | 17 | 5,639,360 |
| 2017 | 19 | 7,497,025 | 7 | 2,297,673 | 19 | 5,648,335 |
| 2018 | 26 | 7,425,917 | 7 | 2,293,434 | 17 | 5,637,862 |

| **Older (≥ 15 years)** | | | | | | |
| --- | --- | --- | --- | --- | --- | --- |
|  | **NHB (N = 131)** | | **NHW (N = 779)** | | **Male (N =184)** | |
| Year | Descents | Population | Descents | Population | Descents | Population |
| 2004 | 9 | 7,207,518 | 52 | 39,311,055 | 11 | 11,621,053 |
| 2005 | 6 | 7,265,401 | 39 | 39,296,693 | 8 | 11,999,309 |
| 2006 | 5 | 7,351,683 | 61 | 39,316,911 | 9 | 12,371,314 |
| 2007 | 12 | 7,486,271 | 39 | 39,347,001 | 15 | 12,747,962 |
| 2008 | 8 | 7,606,024 | 46 | 39,405,194 | 12 | 13,153,969 |
| 2009 | 6 | 7,720,539 | 50 | 39,465,237 | 15 | 13,568,275 |
| 2010 | 5 | 7,822,672 | 51 | 39,520,573 | 13 | 13,958,554 |
| 2011 | 14 | 7,926,518 | 58 | 39,595,506 | 14 | 14,254,220 |
| 2012 | 7 | 8,027,077 | 49 | 39,678,416 | 11 | 14,532,660 |
| 2013 | 7 | 8,118,496 | 54 | 39,718,081 | 7 | 14,811,457 |
| 2014 | 10 | 8,218,542 | 53 | 39,760,933 | 13 | 15,093,860 |
| 2015 | 8 | 8,324,012 | 63 | 39,785,241 | 12 | 15,388,341 |
| 2016 | 10 | 8,426,444 | 51 | 39,779,930 | 15 | 15,683,590 |
| 2017 | 12 | 8,522,631 | 47 | 39,720,930 | 10 | 15,957,364 |
| 2018 | 12 | 8,607,178 | 66 | 39,623,844 | 19 | 16,218,064 |

**Supplemental Table 5. Trends in number of decedents and population by each age subgroups and sex**

|  | **Female and male (N = 2039)** | | **Female (N = 963)** | | **Male (N =1076)** | |
| --- | --- | --- | --- | --- | --- | --- |
| Year | Descents | Population | Descents | Population | Descents | Population |
| 00 years | 13 | 17,135,620 | 8 | 8,372,508 | 5 | 8,763,112 |
| 01-04 years | 155 | 68,809,631 | 82 | 33,641,608 | 73 | 35,168,023 |
| 05-09 years | 493 | 86,075,380 | 268 | 42,096,629 | 225 | 43,978,751 |
| 10-14 years | 177 | 89,230,626 | 85 | 43,613,002 | 92 | 45,617,624 |
| 15-19 years | 96 | 90,947,338 | 36 | 44,259,330 | 60 | 46,688,008 |
| 20-24 years | 69 | 92,256,879 | 30 | 44,693,209 | 39 | 47,563,670 |
| 25-29 years | 67 | 92,252,250 | 29 | 45,264,810 | 38 | 46,987,440 |
| 30-34 years | 84 | 88,940,577 | 42 | 44,076,482 | 42 | 44,864,095 |
| 35-39 years | 96 | 88,346,511 | 39 | 44,172,270 | 57 | 44,174,241 |
| 40-44 years | 79 | 89,706,483 | 33 | 45,076,508 | 46 | 44,629,975 |
| 45-49 years | 68 | 91,581,870 | 28 | 46,224,012 | 40 | 45,357,858 |
| 50-54 years | 108 | 89,061,272 | 43 | 45,298,461 | 65 | 43,762,811 |
| 55-59 years | 103 | 81,229,547 | 43 | 41,782,937 | 60 | 39,446,610 |
| 60-64 years | 105 | 67,280,188 | 40 | 35,085,824 | 65 | 32,194,364 |
| 65-69 years | 85 | 52,312,825 | 36 | 27,769,066 | 49 | 24,543,759 |
| 70-74 years | 67 | 39,050,644 | 28 | 21,275,697 | 39 | 17,774,947 |
| 75-79 years | 57 | 29,685,609 | 24 | 16,801,859 | 33 | 12,883,750 |
| 80-84 years | 69 | 22,207,435 | 36 | 13,272,159 | 33 | 8,935,276 |
| 85+ years | 48 | 22,086,355 | 33 | 14,630,299 | 15 | 7,456,056 |

**Supplemental Table 6. Trends in number of decedents and population across age groups among brainstem glioma**

|  | **All (N = 1523)** | | **Younger (≤ 14 years) (N = 678)** | | **Older (≥ 15 years) (N =845)** | |
| --- | --- | --- | --- | --- | --- | --- |
| Year | Descents | Population | Descents | Population | Descents | Population |
| 2004 | 100 | 82,055,585 | 48 | 17,631,428 | 52 | 64,424,157 |
| 2005 | 84 | 82,537,476 | 34 | 17,503,724 | 50 | 65,033,752 |
| 2006 | 97 | 83,099,557 | 42 | 17,404,381 | 55 | 65,695,176 |
| 2007 | 84 | 83,810,676 | 36 | 17,402,455 | 48 | 66,408,221 |
| 2008 | 89 | 84,618,783 | 43 | 17,448,105 | 46 | 67,170,678 |
| 2009 | 113 | 85,402,713 | 53 | 17,476,352 | 60 | 67,926,361 |
| 2010 | 117 | 86,139,591 | 63 | 17,500,122 | 54 | 68,639,469 |
| 2011 | 108 | 86,790,832 | 45 | 17,486,158 | 63 | 69,304,674 |
| 2012 | 105 | 87,404,830 | 44 | 17,447,725 | 61 | 69,957,105 |
| 2013 | 102 | 87,981,722 | 45 | 17,419,428 | 57 | 70,562,294 |
| 2014 | 95 | 88,585,507 | 44 | 17,392,953 | 51 | 71,192,554 |
| 2015 | 101 | 89,189,512 | 36 | 17,348,017 | 65 | 71,841,495 |
| 2016 | 116 | 89,764,615 | 56 | 17,313,794 | 60 | 72,450,821 |
| 2017 | 96 | 90,244,431 | 44 | 17,281,852 | 52 | 72,962,579 |
| 2018 | 116 | 90,571,210 | 45 | 17,194,763 | 71 | 73,376,447 |

**Supplemental Table 7. Trends in number of decedents and population by sex groups across age groups among brainstem glioma**

| **Younger (≤ 14 years)** | | | | |
| --- | --- | --- | --- | --- |
|  | **Female (N = 370)** | | **Male (N =308)** | |
| Year | Descents | Population | Descents | Population |
| 2004 | 23 | 8,610,981 | 25 | 9,020,447 |
| 2005 | 21 | 8,550,306 | 13 | 8,953,418 |
| 2006 | 19 | 8,503,488 | 23 | 8,900,893 |
| 2007 | 26 | 8,500,992 | 10 | 8,901,463 |
| 2008 | 22 | 8,525,325 | 21 | 8,922,780 |
| 2009 | 28 | 8,541,776 | 25 | 8,934,576 |
| 2010 | 39 | 8,555,469 | 24 | 8,944,653 |
| 2011 | 25 | 8,551,163 | 20 | 8,934,995 |
| 2012 | 22 | 8,534,232 | 22 | 8,913,493 |
| 2013 | 24 | 8,521,981 | 21 | 8,897,447 |
| 2014 | 25 | 8,508,961 | 19 | 8,883,992 |
| 2015 | 18 | 8,487,571 | 18 | 8,860,446 |
| 2016 | 31 | 8,470,470 | 25 | 8,843,324 |
| 2017 | 25 | 8,453,097 | 19 | 8,828,755 |
| 2018 | 22 | 8,407,935 | 23 | 8,786,828 |

| **Older (≥ 15 years)** | | | | |
| --- | --- | --- | --- | --- |
|  | **Female (N = 361)** | | **Male (N =681)** | |
| Year | Descents | Population | Descents | Population |
| 2004 | 21 | 32,957,849 | 31 | 31,466,308 |
| 2005 | 27 | 33,256,884 | 23 | 31,776,868 |
| 2006 | 32 | 33,582,663 | 23 | 32,112,513 |
| 2007 | 18 | 33,948,457 | 30 | 32,459,764 |
| 2008 | 15 | 34,332,610 | 31 | 32,838,068 |
| 2009 | 21 | 34,711,359 | 39 | 33,215,002 |
| 2010 | 20 | 35,082,262 | 34 | 33,557,207 |
| 2011 | 30 | 35,417,466 | 33 | 33,887,208 |
| 2012 | 25 | 35,736,144 | 36 | 34,220,961 |
| 2013 | 23 | 36,037,173 | 34 | 34,525,121 |
| 2014 | 19 | 36,347,309 | 32 | 34,845,245 |
| 2015 | 31 | 36,664,060 | 34 | 35,177,435 |
| 2016 | 24 | 36,965,306 | 36 | 35,485,515 |
| 2017 | 22 | 37,217,839 | 30 | 35,744,740 |
| 2018 | 33 | 37,425,542 | 38 | 35,950,905 |

**Supplemental Table 8. Trends in number of decedents and population by each age subgroups and sex among brainstem glioma**

|  | **Female and male (N = 1523)** | | **Female (N = 731)** | | **Male (N =792)** | |
| --- | --- | --- | --- | --- | --- | --- |
| Year | Descents | Population | Descents | Population | Descents | Population |
| 00 years | 3 | 17,135,620 | 2 | 8,372,508 | 1 | 8,763,112 |
| 01-04 years | 105 | 68,809,631 | 65 | 33,641,608 | 40 | 35,168,023 |
| 05-09 years | 427 | 86,075,380 | 236 | 42,096,629 | 191 | 43,978,751 |
| 10-14 years | 143 | 89,230,626 | 67 | 43,613,002 | 76 | 45,617,624 |
| 15-19 years | 70 | 90,947,338 | 29 | 44,259,330 | 41 | 46,688,008 |
| 20-24 years | 47 | 92,256,879 | 21 | 44,693,209 | 26 | 47,563,670 |
| 25-29 years | 45 | 92,252,250 | 19 | 45,264,810 | 26 | 46,987,440 |
| 30-34 years | 62 | 88,940,577 | 31 | 44,076,482 | 31 | 44,864,095 |
| 35-39 years | 78 | 88,346,511 | 31 | 44,172,270 | 47 | 44,174,241 |
| 40-44 years | 64 | 89,706,483 | 26 | 45,076,508 | 38 | 44,629,975 |
| 45-49 years | 57 | 91,581,870 | 22 | 46,224,012 | 35 | 45,357,858 |
| 50-54 years | 85 | 89,061,272 | 32 | 45,298,461 | 53 | 43,762,811 |
| 55-59 years | 72 | 81,229,547 | 25 | 41,782,937 | 47 | 39,446,610 |
| 60-64 years | 70 | 67,280,188 | 31 | 35,085,824 | 39 | 32,194,364 |
| 65-69 years | 56 | 52,312,825 | 26 | 27,769,066 | 30 | 24,543,759 |
| 70-74 years | 39 | 39,050,644 | 16 | 21,275,697 | 23 | 17,774,947 |
| 75-79 years | 35 | 29,685,609 | 13 | 16,801,859 | 22 | 12,883,750 |
| 80-84 years | 38 | 22,207,435 | 19 | 13,272,159 | 19 | 8,935,276 |
| 85+ years | 27 | 22,086,355 | 20 | 14,630,299 | 7 | 7,456,056 |

**Supplemental Table 9. Trends in number of brainstem tumor patients and population by each age subgroups and sex**

|  | **Female and male (N = 1588)** | | **Female (N = 747)** | | **Male (N =841)** | |
| --- | --- | --- | --- | --- | --- | --- |
| Year | Patients | Population | Patients | Population | Patients | Population |
| 00 years | 23 | 17,135,620 | 11 | 8,372,508 | 12 | 8,763,112 |
| 01-04 years | 242 | 68,809,631 | 134 | 33,641,608 | 108 | 35,168,023 |
| 05-09 years | 371 | 86,075,380 | 192 | 42,096,629 | 179 | 43,978,751 |
| 10-14 years | 128 | 89,230,626 | 57 | 43,613,002 | 71 | 45,617,624 |
| 15-19 years | 59 | 90,947,338 | 25 | 44,259,330 | 34 | 46,688,008 |
| 20-24 years | 52 | 92,256,879 | 24 | 44,693,209 | 28 | 47,563,670 |
| 25-29 years | 54 | 92,252,250 | 24 | 45,264,810 | 30 | 46,987,440 |
| 30-34 years | 58 | 88,940,577 | 21 | 44,076,482 | 37 | 44,864,095 |
| 35-39 years | 63 | 88,346,511 | 27 | 44,172,270 | 36 | 44,174,241 |
| 40-44 years | 56 | 89,706,483 | 19 | 45,076,508 | 37 | 44,629,975 |
| 45-49 years | 58 | 91,581,870 | 21 | 46,224,012 | 37 | 45,357,858 |
| 50-54 years | 76 | 89,061,272 | 26 | 45,298,461 | 50 | 43,762,811 |
| 55-59 years | 79 | 81,229,547 | 31 | 41,782,937 | 48 | 39,446,610 |
| 60-64 years | 64 | 67,280,188 | 27 | 35,085,824 | 37 | 32,194,364 |
| 65-69 years | 58 | 52,312,825 | 29 | 27,769,066 | 29 | 24,543,759 |
| 70-74 years | 39 | 39,050,644 | 17 | 21,275,697 | 22 | 17,774,947 |
| 75-79 years | 36 | 29,685,609 | 18 | 16,801,859 | 18 | 12,883,750 |
| 80-84 years | 42 | 22,207,435 | 23 | 13,272,159 | 19 | 8,935,276 |
| 85+ years | 30 | 22,086,355 | 21 | 14,630,299 | 9 | 7,456,056 |

**Supplemental Table 10. Trends in number of brainstem tumor patients and population by each age subgroups and sex among brainstem glioma**

|  | **Female and male (N = 1264)** | | **Female (N = 604)** | | **Male (N =660)** | |
| --- | --- | --- | --- | --- | --- | --- |
| Year | Patients | Population | Patients | Population | Patients | Population |
| 00 years | 4 | 17,135,620 | 2 | 8,372,508 | 2 | 8,763,112 |
| 01-04 years | 180 | 68,809,631 | 107 | 33,641,608 | 73 | 35,168,023 |
| 05-09 years | 335 | 86,075,380 | 177 | 42,096,629 | 158 | 43,978,751 |
| 10-14 years | 106 | 89,230,626 | 50 | 43,613,002 | 56 | 45,617,624 |
| 15-19 years | 53 | 90,947,338 | 23 | 44,259,330 | 30 | 46,688,008 |
| 20-24 years | 42 | 92,256,879 | 19 | 44,693,209 | 23 | 47,563,670 |
| 25-29 years | 36 | 92,252,250 | 17 | 45,264,810 | 19 | 46,987,440 |
| 30-34 years | 52 | 88,940,577 | 19 | 44,076,482 | 33 | 44,864,095 |
| 35-39 years | 52 | 88,346,511 | 23 | 44,172,270 | 29 | 44,174,241 |
| 40-44 years | 46 | 89,706,483 | 15 | 45,076,508 | 31 | 44,629,975 |
| 45-49 years | 50 | 91,581,870 | 17 | 46,224,012 | 33 | 45,357,858 |
| 50-54 years | 60 | 89,061,272 | 19 | 45,298,461 | 41 | 43,762,811 |
| 55-59 years | 61 | 81,229,547 | 23 | 41,782,937 | 38 | 39,446,610 |
| 60-64 years | 49 | 67,280,188 | 24 | 35,085,824 | 25 | 32,194,364 |
| 65-69 years | 43 | 52,312,825 | 19 | 27,769,066 | 24 | 24,543,759 |
| 70-74 years | 26 | 39,050,644 | 11 | 21,275,697 | 15 | 17,774,947 |
| 75-79 years | 23 | 29,685,609 | 12 | 16,801,859 | 11 | 12,883,750 |
| 80-84 years | 26 | 22,207,435 | 12 | 13,272,159 | 14 | 8,935,276 |
| 85+ years | 20 | 22,086,355 | 15 | 14,630,299 | 5 | 7,456,056 |

**Supplemental Table 11. Trends in age-adjusted mortality rate in brainstem tumors by race/ethnic groups across age groups**

| **Younger (≤ 14 years)** | | | | | | |
| --- | --- | --- | --- | --- | --- | --- |
|  | **NHB (N = 130)** | | **NHW (N = 352)** | | **Hispanic (N =278)** | |
| Year | AAMR  (95% CI) | AAPC  (95% CI) | AAMR  (95% CI) | AAPC  (95% CI) | AAMR  (95% CI) | AAPC  (95% CI) |
| 2004 | 0.57 (0.31, 0.97) | -0.7  (-10.1, 10.8) | 0.14 (0.07, 0.25) | 1.0  (-4.6, 7.2) | 0.36 (0.21, 0.58) | -0.7  (-5.9, 5.3) |
| 2005 | 0.16 (0.04, 0.42) |  | 0.33 (0.22, 0.48) |  | 0.20 (0.09, 0.36) |  |
| 2006 | 0.56 (0.30, 0.95) |  | 0.30 (0.19, 0.44) |  | 0.32 (0.18, 0.51) |  |
| 2007 | 0.04 (0.00, 0.24) |  | 0.32 (0.21, 0.47) |  | 0.34 (0.20, 0.54) |  |
| 2008 | 0.39 (0.18, 0.74) |  | 0.22 (0.13, 0.35) |  | 0.47 (0.31, 0.70) |  |
| 2009 | 0.26 (0.10, 0.57) |  | 0.33 (0.21, 0.48) |  | 0.43 (0.28, 0.65) |  |
| 2010 | 0.47 (0.23, 0.84) |  | 0.42 (0.29, 0.58) |  | 0.42 (0.27, 0.63) |  |
| 2011 | 0.48 (0.24, 0.85) |  | 0.38 (0.26, 0.55) |  | 0.27 (0.15, 0.44) |  |
| 2012 | 0.26 (0.09, 0.56) |  | 0.29 (0.19, 0.44) |  | 0.38 (0.23, 0.58) |  |
| 2013 | 0.35 (0.15, 0.68) |  | 0.23 (0.14, 0.37) |  | 0.35 (0.22, 0.55) |  |
| 2014 | 0.39 (0.18, 0.74) |  | 0.30 (0.19, 0.46) |  | 0.45 (0.29, 0.66) |  |
| 2015 | 0.39 (0.18, 0.74) |  | 0.28 (0.17, 0.42) |  | 0.23 (0.12, 0.39) |  |
| 2016 | 0.65 (0.36, 1.07) |  | 0.35 (0.23, 0.51) |  | 0.30 (0.17, 0.48) |  |
| 2017 | 0.31 (0.12, 0.63) |  | 0.26 (0.15, 0.40) |  | 0.33 (0.20, 0.52) |  |
| 2018 | 0.31 (0.12, 0.63) |  | 0.35 (0.23, 0.52) |  | 0.30 (0.18, 0.49) |  |

| **Older (≥ 15 years)** | | | | | | |
| --- | --- | --- | --- | --- | --- | --- |
|  | **NHB (N = 131)** | | **NHW (N = 779)** | | **Hispanic (N =184)** | |
| Year | AAMR  (95% CI) | AAPC  (95% CI) | AAMR  (95% CI) | AAPC  (95% CI) | AAMR  (95% CI) | AAPC  (95% CI) |
| 2004 | 0.11 (0.05, 0.22) | 1.6  (-5.4, 10.8) | 0.13 (0.09, 0.17) | 1.1  (-2.9, 6.1) | 0.09 (0.04, 0.18) | -1.1  (-8.3, 7.8) |
| 2005 | 0.08 (0.03, 0.18) |  | 0.09 (0.06, 0.12) |  | 0.11 (0.04, 0.23) |  |
| 2006 | 0.09 (0.03, 0.21) |  | 0.15 (0.11, 0.19) |  | 0.09 (0.04, 0.18) |  |
| 2007 | 0.16 (0.08, 0.29) |  | 0.09 (0.06, 0.12) |  | 0.13 (0.07, 0.23) |  |
| 2008 | 0.11 (0.04, 0.22) |  | 0.12 (0.09, 0.16) |  | 0.14 (0.06, 0.25) |  |
| 2009 | 0.08 (0.03, 0.19) |  | 0.13 (0.09, 0.17) |  | 0.11 (0.06, 0.19) |  |
| 2010 | 0.08 (0.03, 0.18) |  | 0.12 (0.09, 0.16) |  | 0.13 (0.06, 0.23) |  |
| 2011 | 0.19 (0.10, 0.33) |  | 0.14 (0.10, 0.18) |  | 0.12 (0.06, 0.21) |  |
| 2012 | 0.08 (0.03, 0.17) |  | 0.12 (0.09, 0.16) |  | 0.07 (0.04, 0.14) |  |
| 2013 | 0.08 (0.03, 0.17) |  | 0.13 (0.10, 0.17) |  | 0.05 (0.02, 0.11) |  |
| 2014 | 0.12 (0.06, 0.23) |  | 0.12 (0.09, 0.16) |  | 0.09 (0.05, 0.17) |  |
| 2015 | 0.11 (0.04, 0.21) |  | 0.15 (0.12, 0.20) |  | 0.09 (0.04, 0.16) |  |
| 2016 | 0.13 (0.06, 0.23) |  | 0.11 (0.08, 0.15) |  | 0.12 (0.06, 0.20) |  |
| 2017 | 0.14 (0.07, 0.25) |  | 0.11 (0.08, 0.15) |  | 0.07 (0.03, 0.13) |  |
| 2018 | 0.16 (0.08, 0.28) |  | 0.15 (0.12, 0.20) |  | 0.12 (0.07, 0.19) |  |

AAMR indicates age-adjusted mortality rate; AAPC, average annual percent change; CI, confidence interval; NHB, non-Hispanic Black; NHW, non-Hispanic White.

**Supplemental Table 12. Total annual age-adjusted mortality rates for brainstem tumors by each age subgroups and sex**

|  | **AAMR (95% CI)** | | **Rate ratio (95% CI)** | **p value** |
| --- | --- | --- | --- | --- |
| **Age group**  **(years)** | **Female** | **Male** |  |  |
| 00 | 0.10 (0.04, 0.19) | 0.06 (0.02, 0.13) | 1.67 (0.48, 6.51) | 0.525 |
| 01-04 | 0.24 (0.19, 0.30) | 0.21 (0.16, 0.26) | 1.17 (0.85, 1.63) | 0.358 |
| 05-09 | 0.64 (0.56, 0.72) | 0.51 (0.45, 0.58) | 1.24 (1.04, 1.49) | 0.017* |
| 10-14 | 0.19 (0.16, 0.24) | 0.20 (0.16, 0.25) | 0.97 (0.71, 1.31) | 0.880 |
| 15-19 | 0.08 (0.06, 0.11) | 0.13 (0.10, 0.17) | 0.63 (0.41, 0.97) | 0.036* |
| 20-24 | 0.07 (0.05, 0.10) | 0.08 (0.06, 0.11) | 0.82 (0.49, 1.35) | 0.482 |
| 25-29 | 0.06 (0.04, 0.09) | 0.08 (0.06, 0.11) | 0.79 (0.47, 1.32) | 0.410 |
| 30-34 | 0.10 (0.07, 0.13) | 0.09 (0.07, 0.13) | 1.02 (0.65, 1.60) | 1.000 |
| 35-39 | 0.09 (0.06, 0.12) | 0.13 (0.10, 0.17) | 0.68 (0.44, 1.05) | 0.082 |
| 40-44 | 0.07 (0.05, 0.10) | 0.10 (0.08, 0.14) | 0.71 (0.44, 1.14) | 0.163 |
| 45-49 | 0.06 (0.04, 0.09) | 0.09 (0.06, 0.12) | 0.69 (0.41, 1.14) | 0.158 |
| 50-54 | 0.09 (0.07, 0.13) | 0.15 (0.11, 0.19) | 0.64 (0.42, 0.95) | 0.005* |
| 55-59 | 0.10 (0.07, 0.14) | 0.15 (0.12, 0.20) | 0.68 (0.45, 1.02) | 0.061 |
| 60-64 | 0.11 (0.08, 0.16) | 0.20 (0.16, 0.26) | 0.56 (0.37, 0.85) | 0.005* |
| 65-69 | 0.13 (0.09, 0.18) | 0.20 (0.15, 0.26) | 0.65 (0.41, 1.02) | 0.061 |
| 70-74 | 0.13 (0.09, 0.19) | 0.22 (0.16, 0.30) | 0.60 (0.36, 1.00) | 0.05 |
| 75-79 | 0.14 (0.09, 0.21) | 0.26 (0.18, 0.36) | 0.56 (0.32, 0.97) | 0.039* |
| 80-84 | 0.27 (0.19, 0.38) | 0.37 (0.25, 0.52) | 0.73 (0.45, 1.22) | 0.246 |
| > 85 | 0.23 (0.16, 0.32) | 0.20 (0.11, 0.33) | 1.12 (0.59, 2.22) | 0.843 |

AAMR indicates age-adjusted mortality rate; CI, confidence interval. Statistical significance was calculated with SEER*Stat software.

*p < 0.05

**Supplemental Table 13. Total annual age-adjusted incidence rates for brainstem tumors by each age subgroups and sex**

|  | **AAIR (95% CI)** | | **Rate ratio (95% CI)** | **p value** |
| --- | --- | --- | --- | --- |
| **Age group**  **(years)** | **Female** | **Male** |  |  |
| 00 | 0.13 (0.07, 0.24) | 0.14 (0.07, 0.24) | 0.96 (0.38, 2.38) | 1.000 |
| 01-04 | 0.40 (0.33, 0.47) | 0.31 (0.25, 0.37) | 1.30 (1.00, 1.69) | 0.051 |
| 05-09 | 0.46 (0.39, 0.53) | 0.41 (0.35, 0.47) | 1.12 (0.91, 1.38) | 0.296 |
| 10-14 | 0.13 (0.10, 0.17) | 0.16 (0.12, 0.20) | 0.84 (0.58, 1.21) | 0.371 |
| 15-19 | 0.06 (0.04, 0.08) | 0.07 (0.05, 0.10) | 0.78 (0.44, 1.34) | 0.403 |
| 20-24 | 0.05 (0.03, 0.08) | 0.06 (0.04, 0.09) | 0.91 (0.51, 1.63) | 0.849 |
| 25-29 | 0.05 (0.03, 0.08) | 0.06 (0.04, 0.09) | 0.83 (0.46, 1.47) | 0.588 |
| 30-34 | 0.05 (0.03, 0.07) | 0.08 (0.06, 0.11) | 0.58 (0.32, 1.01) | 0.056 |
| 35-39 | 0.06 (0.04, 0.09) | 0.08 (0.06, 0.11) | 0.75 (0.44, 1.27) | 0.314 |
| 40-44 | 0.04 (0.03, 0.07) | 0.08 (0.06, 0.11) | 0.51 (0.28, 0.91) | 0.020* |
| 45-49 | 0.05 (0.03, 0.07) | 0.08 (0.06, 0.11) | 0.56 (0.31, 0.98) | 0.040* |
| 50-54 | 0.06 (0.04, 0.08) | 0.11 (0.08, 0.15) | 0.50 (0.30, 0.82) | 0.005* |
| 55-59 | 0.07 (0.05, 0.11) | 0.12 (0.09, 0.16) | 0.61 (0.38, 0.98) | 0.039* |
| 60-64 | 0.08 (0.05, 0.11) | 0.11 (0.08, 0.16) | 0.67 (0.39, 1.13) | 0.142 |
| 65-69 | 0.10 (0.07, 0.15) | 0.12 (0.08, 0.17) | 0.88 (0.51, 1.53) | 0.733 |
| 70-74 | 0.08 (0.05, 0.13) | 0.12 (0.08, 0.19) | 0.65 (0.32, 1.27) | 0.229 |
| 75-79 | 0.11 (0.06, 0.17) | 0.14 (0.08, 0.22) | 0.77 (0.38, 1.56) | 0.526 |
| 80-84 | 0.17 (0.11, 0.26) | 0.21 (0.13, 0.33) | 0.82 (0.42, 1.58) | 0.610 |
| > 85 | 0.14 (0.09, 0.22) | 0.12 (0.06, 0.23) | 1.19 (0.52, 2.95) | 0.825 |

AAIR indicates age-adjusted incident rate; CI, confidence interval. Statistical significance was calculated with SEER*Stat software.

*p < 0.05

**Supplemental Table 14. Total annual age-adjusted mortality rates for brainstem glioma by each age subgroups and sex**

|  | **AAMR (95% CI)** | | **Rate ratio (95% CI)** | **p value** |
| --- | --- | --- | --- | --- |
| **Age group**  **(years)** | **Female** | **Male** |  |  |
| 00 | 0.02 (0.00, 0.09) | 0.01 (0.00, 0.06) | 2.09 (0.11, 123.50) | 0.966 |
| 01-04 | 0.19 (0.15, 0.25) | 0.11 (0.08, 0.15) | 1.70 (1.13, 2.59) | 0.010* |
| 05-09 | 0.56 (0.49, 0.64) | 0.43 (0.37, 0.50) | 1.29 (1.06, 1.57) | 0.010* |
| 10-14 | 0.15 (0.12, 0.20) | 0.17 (0.13, 0.21) | 0.92 (0.65, 1.30) | 0.689 |
| 15-19 | 0.07 (0.04, 0.09) | 0.09 (0.06, 0.12) | 0.75 (0.45, 1.23) | 0.275 |
| 20-24 | 0.05 (0.03, 0.07) | 0.05 (0.04, 0.08) | 0.86 (0.46, 1.59) | 0.713 |
| 25-29 | 0.04 (0.03, 0.07) | 0.06 (0.04, 0.08) | 0.76 (0.40, 1.42) | 0.442 |
| 30-34 | 0.07 (0.05, 0.10) | 0.07 (0.05, 0.10) | 1.02 (0.60, 1.73) | 1.000 |
| 35-39 | 0.07 (0.05, 0.10) | 0.11 (0.08, 0.14) | 0.66 (0.41, 1.06) | 0.089 |
| 40-44 | 0.06 (0.04, 0.08) | 0.09 (0.06, 0.12) | 0.68 (0.39, 1.15) | 0.157 |
| 45-49 | 0.05 (0.03, 0.07) | 0.08 (0.05, 0.11) | 0.62 (0.34, 1.08) | 0.096 |
| 50-54 | 0.07 (0.05, 0.10) | 0.12 (0.09, 0.16) | 0.58 (0.36, 0.92) | 0.020* |
| 55-59 | 0.06 (0.04, 0.09) | 0.12 (0.09, 0.16) | 0.50 (0.30, 0.83) | 0.006* |
| 60-64 | 0.09 (0.06, 0.13) | 0.12 (0.09, 0.17) | 0.73 (0.44, 1.20) | 0.231 |
| 65-69 | 0.09 (0.06, 0.14) | 0.12 (0.08, 0.17) | 0.77 (0.44, 1.34) | 0.387 |
| 70-74 | 0.08 (0.04, 0.12) | 0.13 (0.08, 0.19) | 0.58 (0.29, 1.15) | 0.127 |
| 75-79 | 0.08 (0.04, 0.13) | 0.17 (0.11, 0.26) | 0.45 (0.21, 0.94) | 0.032* |
| 80-84 | 0.14 (0.09, 0.22) | 0.21 (0.13, 0.33) | 0.67 (0.34, 1.34) | 0.289 |
| > 85 | 0.14 (0.08, 0.21) | 0.09 (0.04, 0.19) | 1.46 (0.59, 4.08) | 0.520 |

AAMR indicates age-adjusted mortality rate; CI, confidence interval. Statistical significance was calculated with SEER*Stat software.

*p < 0.05

**Supplemental Table 15. Total annual age-adjusted incidence rates for brainstem glioma by each age subgroups and sex**

|  | **AAIR (95% CI)** | | **Rate ratio (95% CI)** | **p value** |
| --- | --- | --- | --- | --- |
| **Age group**  **(years)** | **Female** | **Male** |  |  |
| 00 | 0.02 (0.00, 0.09) | 0.02 (0.00, 0.08) | 1.05 (0.08, 14.44) | 1.000 |
| 01-04 | 0.32 (0.26, 0.38) | 0.21 (0.16, 0.26) | 1.53 (1.13, 2.09) | 0.006* |
| 05-09 | 0.42 (0.36, 0.49) | 0.36 (0.31, 0.42) | 1.17 (0.94, 1.46) | 0.166 |
| 10-14 | 0.11 (0.09, 0.15) | 0.12 (0.09, 0.16) | 0.93 (0.62, 1.39) | 0.800 |
| 15-19 | 0.05 (0.03, 0.08) | 0.06 (0.04, 0.09) | 0.81 (0.45, 1.44) | 0.530 |
| 20-24 | 0.04 (0.03, 0.07) | 0.05 (0.03, 0.07) | 0.88 (0.45, 1.69) | 0.795 |
| 25-29 | 0.04 (0.02, 0.06) | 0.04 (0.02, 0.06) | 0.93 (0.45, 1.89) | 0.957 |
| 30-34 | 0.04 (0.03, 0.07) | 0.07 (0.05, 0.10) | 0.59 (0.31, 1.06) | 0.081 |
| 35-39 | 0.05 (0.03, 0.08) | 0.07 (0.04, 0.09) | 0.79 (0.44, 1.42) | 0.489 |
| 40-44 | 0.03 (0.02, 0.05) | 0.07 (0.05, 0.10) | 0.48 (0.24, 0.91) | 0.024* |
| 45-49 | 0.04 (0.02, 0.06) | 0.07 (0.05, 0.10) | 0.51 (0.26, 0.93) | 0.028* |
| 50-54 | 0.04 (0.03, 0.07) | 0.09 (0.07, 0.13) | 0.45 (0.25, 0.79) | 0.004* |
| 55-59 | 0.06 (0.03, 0.08) | 0.10 (0.07, 0.13) | 0.57 (0.33, 0.98) | 0.043* |
| 60-64 | 0.07 (0.04, 0.10) | 0.08 (0.05, 0.11) | 0.88 (0.48, 1.61) | 0.762 |
| 65-69 | 0.07 (0.04, 0.11) | 0.10 (0.06, 0.15) | 0.70 (0.36, 1.33) | 0.310 |
| 70-74 | 0.05 (0.03, 0.09) | 0.08 (0.05, 0.14) | 0.61 (0.25, 1.43) | 0.294 |
| 75-79 | 0.07 (0.04, 0.12) | 0.09 (0.04, 0.15) | 0.84 (0.34, 2.09) | 0.821 |
| 80-84 | 0.09 (0.05, 0.16) | 0.16 (0.09, 0.26) | 0.58 (0.24, 1.34) | 0.226 |
| > 85 | 0.10 (0.06, 0.17) | 0.07 (0.02, 0.16) | 1.53 (0.53, 5.38) | 0.567 |

AAIR indicates age-adjusted incident rate; CI, confidence interval. Statistical significance was calculated with SEER*Stat software.

*p < 0.05


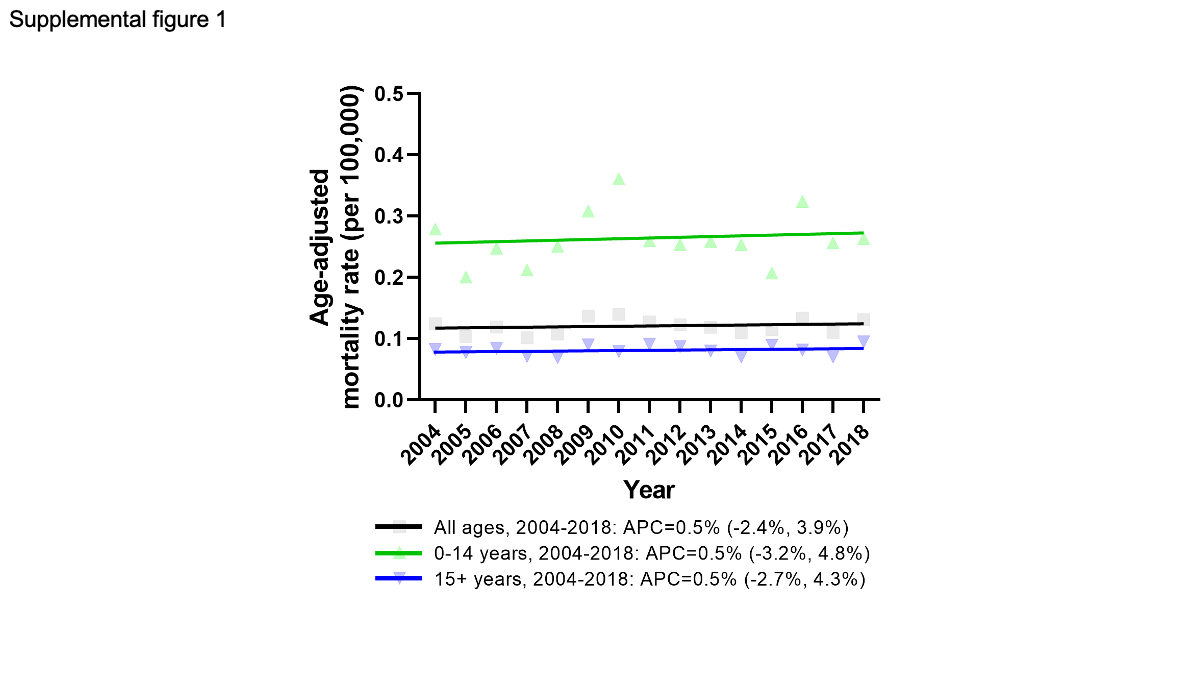


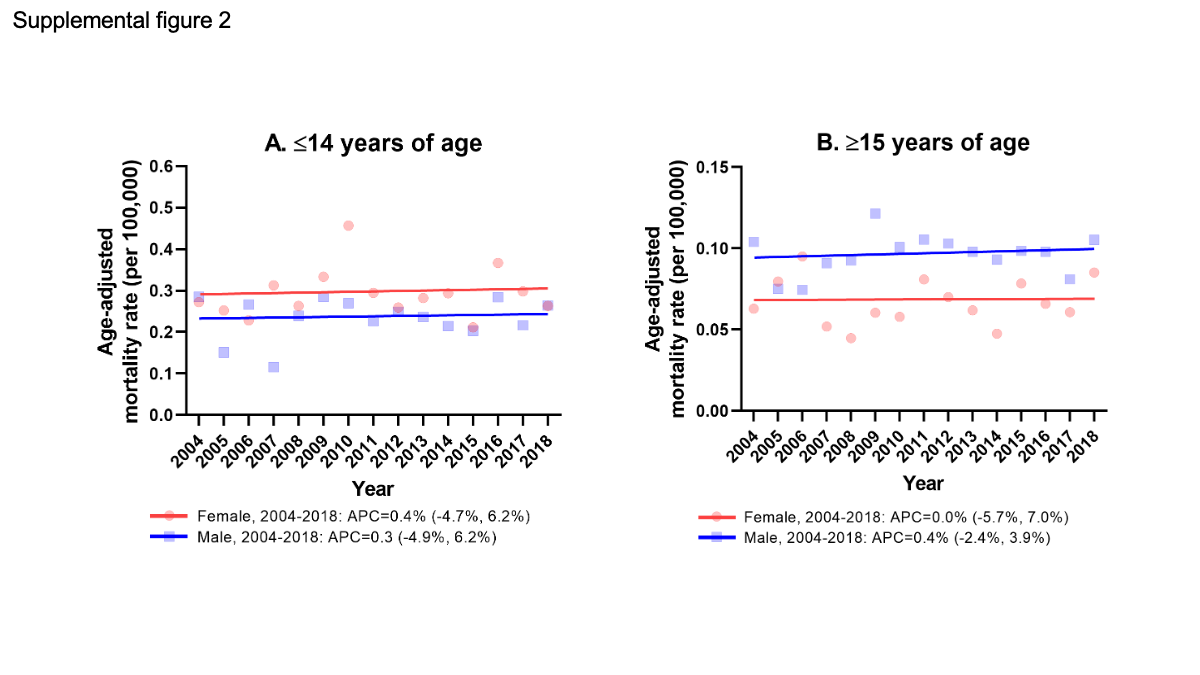


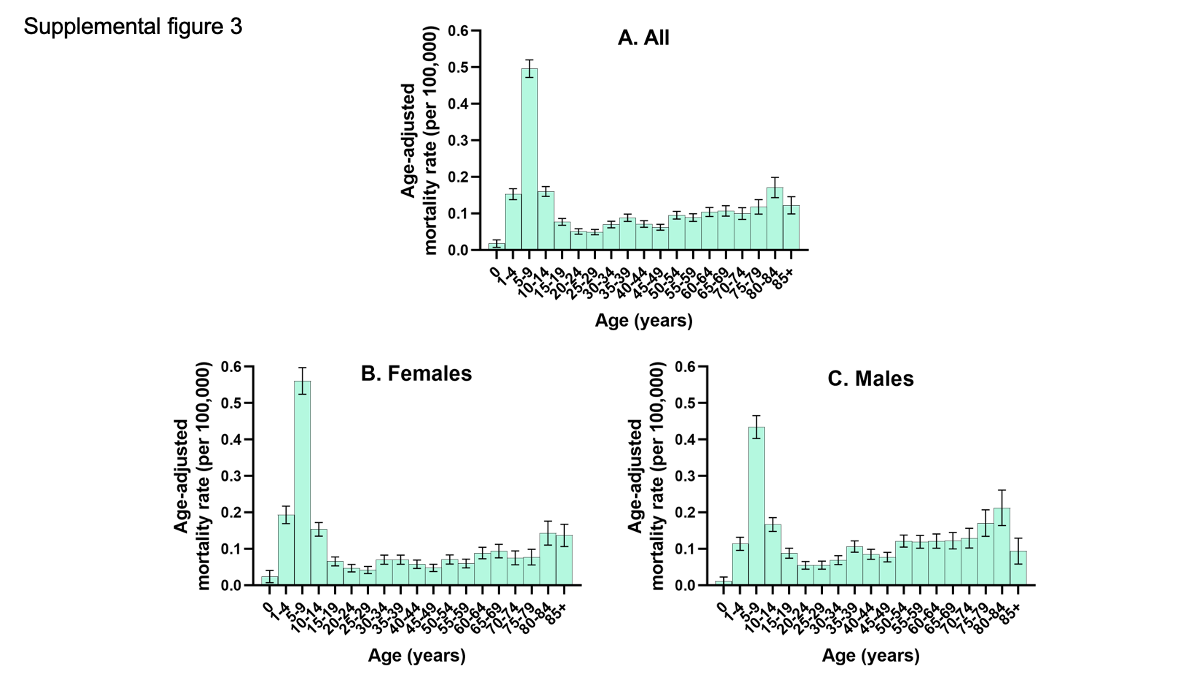

Supplement: vdab137_suppl_Supplementary_File [file vdab137_suppl_supplementary_file.docx]
